# Recovery and Quantification of Norovirus in Air Samples from Experimentally Produced Aerosols

**DOI:** 10.1007/s12560-024-09590-7

**Published:** 2024-03-21

**Authors:** Kitwadee Rupprom, Yuwanda Thongpanich, Woravat Sukkham, Fuangfa Utrarachkij, Leera Kittigul

**Affiliations:** 1 Department of Clinical Pathology, Faculty of Medicine Vajira Hospital, Navamindradhiraj University, Bangkok, Thailand; 2 Department of Microbiology, Faculty of Public Health, Mahidol University, 420/1 Ratchawithi Road, Bangkok, 10400 Thailand

**Keywords:** Norovirus, Aerosol, SpeedVac centrifugation, Biosampler, Virus recovery

## Abstract

Norovirus is the leading cause of acute gastroenteritis in humans across all age groups worldwide. Norovirus-infected patients can produce aerosolized droplets which play a role in gastroenteritis transmission. The study aimed to assess bioaerosol sampling in combination with a virus concentrating procedure to facilitate molecular detection of norovirus genogroup (G) II from experimentally contaminated aerosols. Using a nebulizer within an experimental chamber, aerosols of norovirus GII were generated at known concentrations. Air samples were then collected in both 5 mL and 20 mL water using the SKC BioSampler at a flow rate of 12.5 L/min, 15 min. Subsequently, the virus in collected water was concentrated using speedVac centrifugation and quantified by RT-qPCR. The optimal distances between the nebulizer and the SKC BioSampler yielded high recoveries of the virus for both 5 and 20 mL collections. Following nebulization, norovirus GII RNA was detectable up to 120 min in 5 mL and up to 240 min in 20 mL collection. The concentrations of norovirus GII RNA recovered from air samples in the aerosol chamber ranged from 10^2^ to 10^5^ genome copies/mL, with average recoveries of 25 ± 12% for 5 mL and 22 ± 19% for 20 mL collections. These findings provide quantitative data on norovirus GII in aerosols and introduce a novel virus concentrating method for aerosol collection in water, thus enhancing surveillance of this virus.

## Introduction

Human noroviruses are a major cause of acute gastroenteritis, accounting for one‐fifth of all gastroenteritis cases worldwide (Lopman et al., 2016). These viruses affect people of all ages with increased infection rates occurring in neonates, the elderly, and immunocompromised patients (Bok & Green, 2012; Ottosson et al., 2022). Noroviruses, nonenveloped single-stranded RNA viruses, belong to the *Caliciviridae* family and are divided into 10 genogroups, each further subdivided into genotypes. Genogroups I and II are responsible for the majority of human gastroenteritis, with genogroup II genotype 4 (GII.4) being the most prevalent globally (Chhabra et al., 2019).

Transmission of norovirus via the fecal–oral route primarily occurs from person‐to‐person and through contaminated food and water. Person-to-person spread may occur directly through ingestion of aerosolized vomitus or indirectly via contact with contaminated environmental surfaces (de Graaf et al., 2016). Noroviruses are highly contagious with a low infectious dose, estimated to be 18–1000 viral particles (Atmar et al., 2014; Teunis et al., 2008) and have been proposed to spread opportunistically via droplet nuclei or aerosol transmission (La Rosa et al., 2013). The possibility of airborne transmission of norovirus has been documented in various settings including hospitals (Alsved et al., 2020a), kindergartens (Zhang et al., 2017), and healthcare facilities (Bonifait et al., 2015) where acute gastroenteritis outbreaks have occurred. Vomiting may result in environmental contamination, leading to transmission through fomites and airborne droplets; therefore, aerosols and droplets from vomiting could be a source of transmission (Hagbom et al., 2021; Kirby et al., 2016). Toilet flushing can aerosolize norovirus that may be subsequently inhaled, settled in the upper respiratory tract, and contribute to gastroenteritis when swallowed (Boles et al., 2021).

The importance of aerosols in the spread of norovirus has been continuously studied. Since norovirus is present in air with low density, evaluations of air sampling collection and detection methods have been performed to assess of viral infectivity after aerosolization in a chamber, using murine norovirus as a surrogate for the human norovirus (Alsved et al., 2020b; Boles et al., 2020; Uhrbrand et al., 2018). Among air sampling devices (impactors, liquid impingers, and filters), the SKC BioSampler is the most widely used liquid-based impinger, considered to maintain viral infectivity more effectively than other air sampling methods (Bhardwaj et al., 2021). However, viruses are collected in relatively large volumes, such as 5 and 20 mL of liquid in the collection vessels used in the SKC BioSampler. Virus measurement using cultivation techniques for human norovirus may be difficult in a tissue culture system (Ettayebi et al., 2016). SpeedVac centrifugation with vacuum has been used to reduce the volume of water and concentrate norovirus efficiently, which was subsequently detected in water samples using a highly sensitive molecular method (Kittigul et al., 2019).

Assessing the potential role of norovirus in airborne transmission has been hindered by differences in the experimental sampling and detection methods. In this study, we improved a method to concentrate the aerosolized human norovirus GII, collected using the liquid impinger from an aerosol chamber capable of generating virus as aerosols under laboratory conditions. The aerosols were collected by the SKC BioSampler in water of 5 and 20 mL, which were further reduced in volume using speedVac centrifugation. Subsequently, the virus in the concentrate was detected using the quantitative reverse transcription-polymerase chain reaction (RT-qPCR).

## Materials and Methods

### Aerosol Chamber

**Fig. 1:**
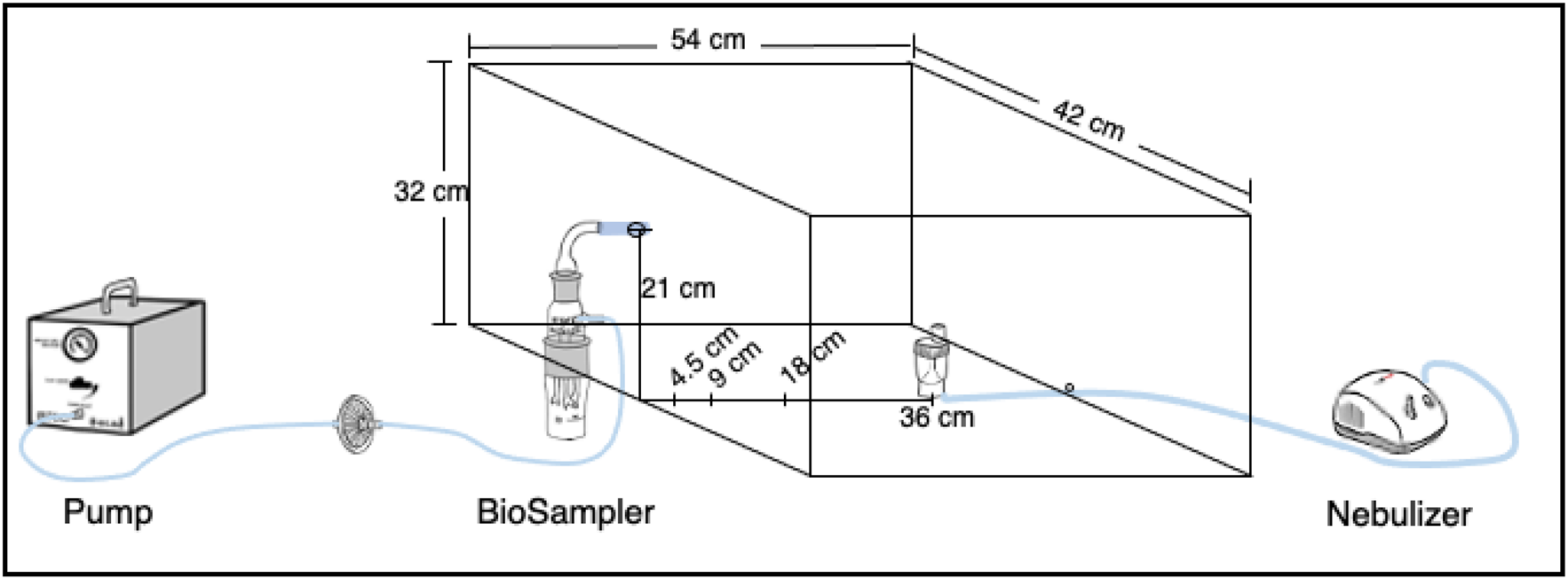
Schematic of the experimental chamber for aerosolization inside a biosafety cabinet and the subsequent analysis of human norovirus GII RNA. The nebulizer served as the aerosol generator, connected through a flow tube to the generator inside the chamber. The aerosols were collected into an SKC BioSampler impinger connected to a suction pump. The collection liquid in the SKC BioSampler was then concentrated and analyzed using RT-qPCR for viral RNA quantification

Experiments were performed inside an acrylic chamber (42 × 54 × 32 cm) placed in a biological safety cabinet (BSC), class II. The Nebulizer NE100 Piston Type (Rossmax Swiss GmbH, Switzerland) with a capacity of 5 mL solution containing norovirus GII-positive stool sample was used to generate viral aerosols at 0.35 mL/min for 15 min. After 15 min of aerosolization, the nebulizer produced aerosols of approximately 4 mL with the remaining 1 mL solution. The SKC BioSampler® (SKC Inc., Eighty-Four, PA, USA), an impinger biosampler, was employed to collect aerosolized material into sterile distilled water of 5 mL and 20 mL collection vessels at a flow rate of 12.5 L/min, 15 min. Figure 1 shows a schematic figure of the established aerosol chamber with the nebulizer and the SKC BioSampler. Virus suspension in the nebulizer and the SKC BioSampler were collected and quantified for norovirus in further laboratory assays.

### Virus Concentration

The virus suspension in the SKC BioSampler (5 and 20 mL) was processed using speedVac centrifugation (Kittigul et al., 2019). The samples were placed in a vacuum centrifuge (UNIEQUIP Laborgeratebau und vertriebs GmbH, Munich, Germany) to reduce the volume to approximately 500 μL for 2 h (5 mL water collection) and for 4 h (20 mL water collection). All concentrated air samples were stored at −80 °C until use for nucleic acid extraction.

### RNA Extraction and RT-qPCR

Virus-containing samples from the nebulizer and the concentrates from the SKC BioSampler (200 μL each) were extracted for RNA using QIAamp® Viral RNA Mini Kit (Qiagen, Hilden, Germany) following the manufacturer's instructions. The extracted RNA was eluted with a final volume of 60 μL. Undiluted and 1:10 diluted RNA extracts were quantified for norovirus GII using RT‐qPCR. The specific primers and probe targeted ORF1-ORF2 overlapping region of norovirus GII as described in ISO/TS 15216-1:2017 (ISO, 2017).

The one-step RT-qPCR was carried out in a 20 μL reaction mixture using the LightCycler® RNA Master Hybprobe with *Tth* DNA polymerase (Roche Diagnostics, Mannheim, Germany), according to the manufacturer’s protocols with some modification (Kittigul et al., 2022). Briefly, RT-qPCR reaction consisted of 1X LightCycler® RNA Master Hybprobe, *Tth* DNA polymerase reaction buffer, dNTP mix (with dUTP instead of dTTP), 3.2 mM Mn(OAc)_2_, 0.5 µM of primer QNIF2, 0.9 µM of primer COG2R, 0.2 µM of probe QNIFs, 5 μL extracted RNA, and nuclease-free water to make the reaction volume up to 20 μL. RT-PCR cycling on the LightCycler® 96 Real-Time PCR instrument (Roche Diagnostics) consisted of 55 °C for 30 min and 95 °C for 5 min, followed by 45 cycles of 95 °C for 15 s and 60 °C for 1 min. Norovirus GII genomic copies were estimated by comparison with a standard curve. The standard curve was established using a dilution series of norovirus GII RNA transcripts plotted against the quantification cycle (Cq) value.

RT-qPCR for norovirus GI was performed using specific primers and a probe (Rupprom et al., 2018). The one-step RT-qPCR was carried out in a 20 μL reaction mixture of the LightCycler® RNA Master Hybprobe with *Tth* DNA polymerase (Roche Diagnostics). Briefly, a 5 μL of the RNA extract was mixed with 15 μL of the RT-qPCR reaction mixture, 3.2 mM Mn(OAc)_2_, 0.4 µM each of primers GITF and GITR, 0.2 µM of probe GIT-TP and nuclease-free water. The thermal cycling conditions were 58 °C for 30 min; 95 °C for 4 min; 45 cycles of 95 °C for 15 s, and 55 °C for 1 min on the LightCycler® 96 Real-Time PCR instrument (Roche Diagnostics). A standard curve for norovirus GI was generated using a dilution series of norovirus GI RNA transcripts plotted against the Cq value.

### Experimental Settings

The starting concentration of norovirus GII, pre-nebulization, was quantified using RT-qPCR. In the established chamber, a norovirus GII suspension (5.5 × 10^6^ genome copies/mL) in sterile distilled water (5 mL) was aerosolized using the Nebulizer NE100 Piston Type. When the nebulizer turned on and produced virus aerosols at 0.35 mL/min for 15 min, an air sampling pump for the SKC BioSampler was operated and calibrated at a flow rate of 12.5 L/min, 15 min. Three experiments were conducted to investigate:

1. The optimal distance within the chamber for collecting aerosol samples from the nebulizer, positioned at 4.5, 9.0, 18 and 36 cm apart from the SKC BioSampler, each distance repeated twice at both 5 and 20 mL collections.
2. The duration of norovirus presence in aerosols at 15, 30, 60, 120, 180, and 240 min. The aerosols were generated from the nebulizer at 36 cm distance for 5 and 20 mL collections. At 15 min duration, sampling commenced simultaneously and concluded within the same 15 min period. At 30 min duration, sampling initiated after aerosol generation (15 min) and concluded within 30 min. Additional experiments of varying duration were performed with the sampling commencing after aerosol generation (15 min) at 45, 105, 165, and 225 min, concluding within 60, 120, 180, and 240 min, respectively. The starting concentrations of norovirus GII in 1) and 2) were in the range of 10^5^ − 10^6^ genome copies/mL.
3. The norovirus concentrations in aerosols, collected using the SKC BioSampler that could be determined by RT-qPCR. In 12 trials, various concentrations of norovirus GII were spiked in the nebulizer ranging from 10^3^ to 10^6^ genome copies/mL. The generated aerosols were collected and quantified for recovered norovirus GII.

Norovirus GII genome copies in the generated aerosols were determined by subtracting the remaining concentrations in the nebulizer from the initial norovirus concentrations, termed as aerosolized norovirus. The aerosols, collected in water during sampling, were concentrated using speedVac centrifugation and then assessed for norovirus concentrations, termed as recovered norovirus.

Virus recovery was calculated as a percentage of the genome copies obtained from the recovered norovirus, divided by genome copies of the aerosolized norovirus. The SKC BioSampler collected aerosolized norovirus GII into sterile distilled water in 5 and 20 mL collection vessels at a flow rate of 12.5 L/min, 15 min (equal to 187.5 L). This collected air volume was then extrapolated to 1000 L or 1 cubic metre (m^3^), and the concentrations of norovirus GII RNA were calculated based on the amount of genome copies/m^3^ of air volume.

### RT-PCR Inhibition Test

RT-PCR inhibition for noroviruses GI and GII was tested in aerosol control collection in the chamber without spiked norovirus in triplicate experiments. The nebulizer containing distilled water was used to produce aerosols which were then collected by the SKC BioSampler and concentrated by speedVac centrifugation. The water concentrate of aerosol control was extracted for RNA. A 1 μL aliquot of norovirus GI or GII RNA transcript (10^4^ RNA copies/μL), named external control (EC) was added to 5 μL of the aerosol control RNA or 5 μL of PCR grade water. RT-qPCR was performed for noroviruses GI and GII in separate tubes. RT-PCR inhibition was calculated according to the following equation: RT-PCR inhibition = (1 − 10^ΔCq/m^) × 100% where ΔCq = Cq value (aerosol control RNA + EC RNA) − Cq value (water + EC RNA) and m = slope of the standard curve. RT-PCR inhibition ≤ 75% was the acceptable level for result analysis (ISO, 2017).

### Statistical Analysis

The differences in norovirus GII RNA recovery in 5 mL and 20 mL collections were determined using the Mann–Whitney U test. The paired t-test was employed to test the differences in RT-PCR inhibition between undiluted RNA and 1:10 diluted RNA extracts for noroviruses GI and GII in 5 and 20 mL sampling. A *p*-value < 0.05 was considered to be statistically significant. Statistical analyses were done using SPSS statistics version 28.0 for Windows (IBM Corp., Armonk, NY, USA).

## Results

### Distance between Nebulizer and SKC BioSampler in the Aerosol Chamber

**Fig. 2:**
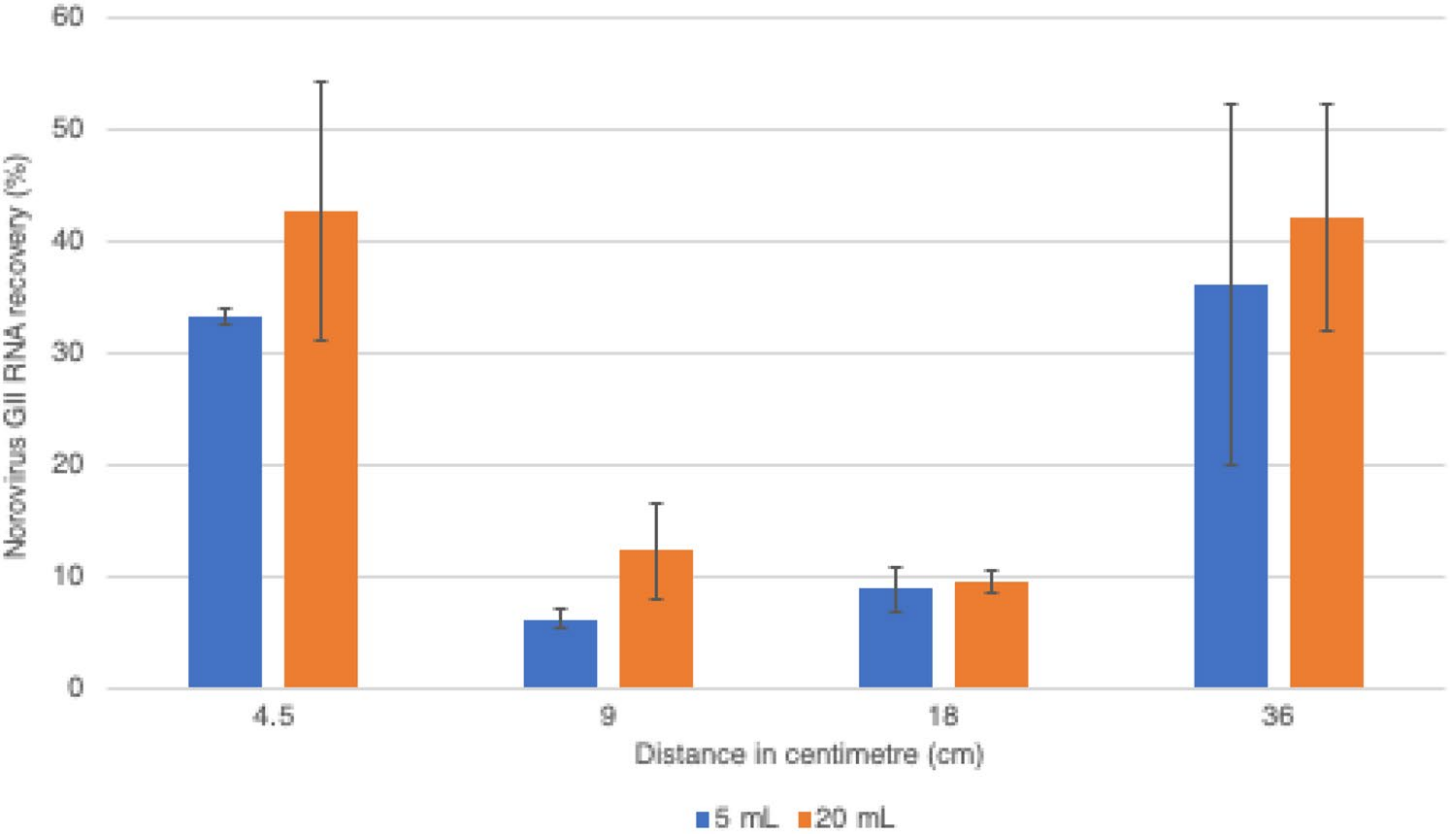
Recovery of norovirus GII RNA in experimentally produced aerosols within the chamber, generated using a nebulizer placed at varying distances from the SKC BioSampler, with both 5 and 20 mL collections (see legend). The experiments were performed in duplicates

During experiments, air samples were collected in the aerosol chamber with temperatures in a range of 23.9 − 24.8 °C and relative humidity of 48 − 54%. The distances between the nebulizer, used to generate aerosols, and the SKC BioSampler, utilized to collect the aerosols in water, were optimized. The average recovery of viral RNA was more efficient at both the closer (4.5 cm) and the farther (36 cm) distances between the nebulizer and the SKC BioSampler in the chamber (33% for 5 mL collection and 43% for 20 mL collection at 4.5 cm, 36% for 5 mL collection and 42% for 20 mL collection at 36 cm) (Fig. 2). The nebulizer placed at a 36 cm distance and the SKC BioSampler operated at a flow rate of 12.5 L/min, 15 min were used in further experiments.

### Norovirus GII Lasting in Aerosols

**Fig. 3:**
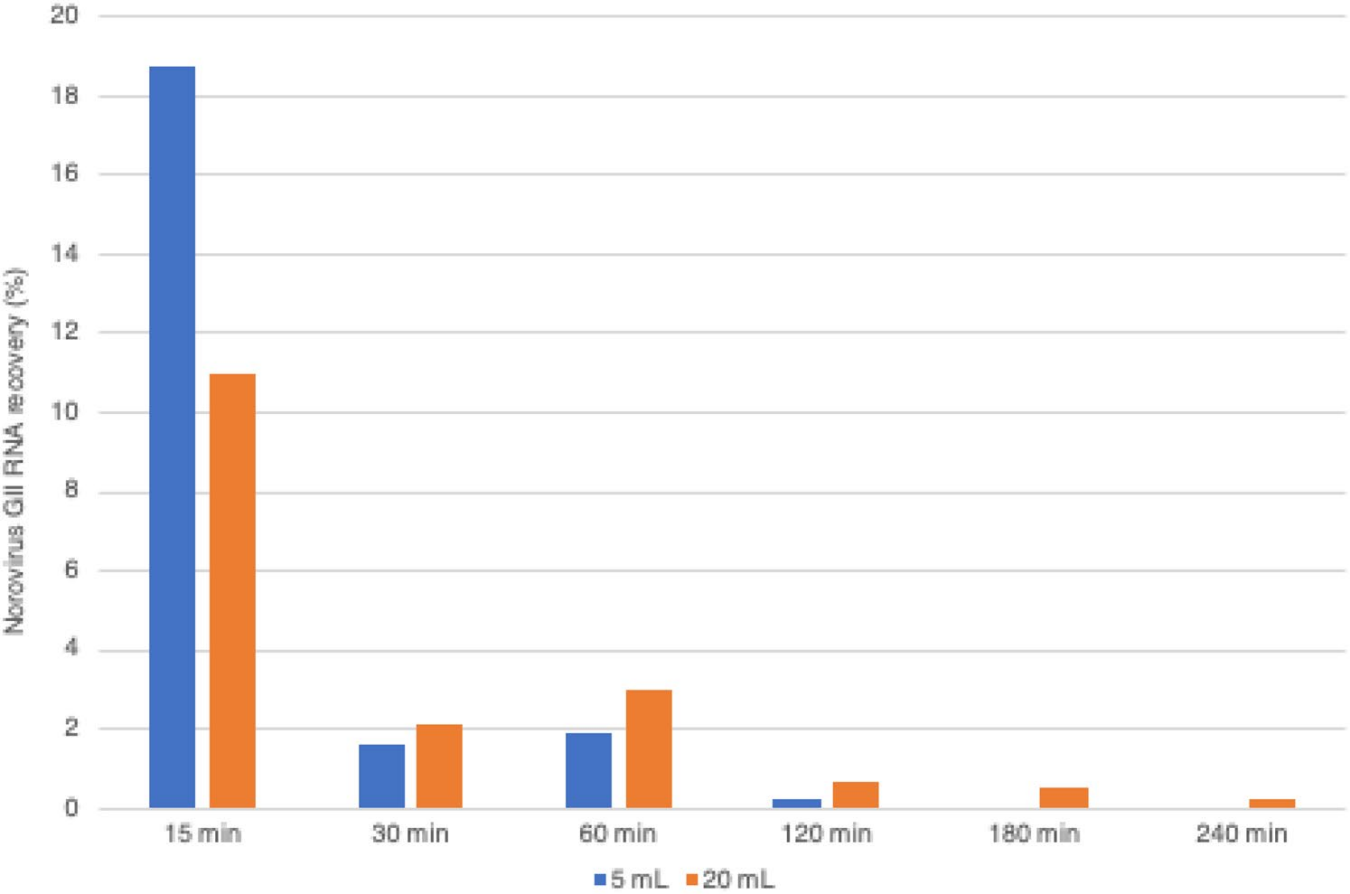
Recovery of norovirus GII RNA lasting in aerosols generated by the nebulizer, placed at 36 cm distance from the SKC BioSampler, with 5 and 20 mL collections at different air sampling durations (see legend)

Norovirus GII RNA present in aerosols was studied in experiments with different collection time-periods. The concentrations of norovirus were gradually decreased in aerosols obtained in both 5 and 20 mL collection vessels of the SKC BioSampler with longer aerosol times. For 5 mL water collection, the virus recovery was 18.7% at 15 min, 1.6% at 30 min, 1.9% at 60 min, and 0.2% at 120 min. Norovirus GII RNA could not be recovered after 2 h from aerosols produced in the chamber. For 20 mL water collection, the virus recovery was 11.0% at 15 min, 2.1% at 30 min, 3.0% at 60 min, 0.7% at 120 min, 0.5% at 180 min, and 0.2% at 240 min. Of note, the concentrations of norovirus GII RNA obtained in the 20 mL collection vessel of the SKC BioSampler could be detected at all aerosol time-periods up to 4 h (Fig. 3).

### Norovirus GII Concentrations and Recovery from Aerosols

**Fig. 4:**
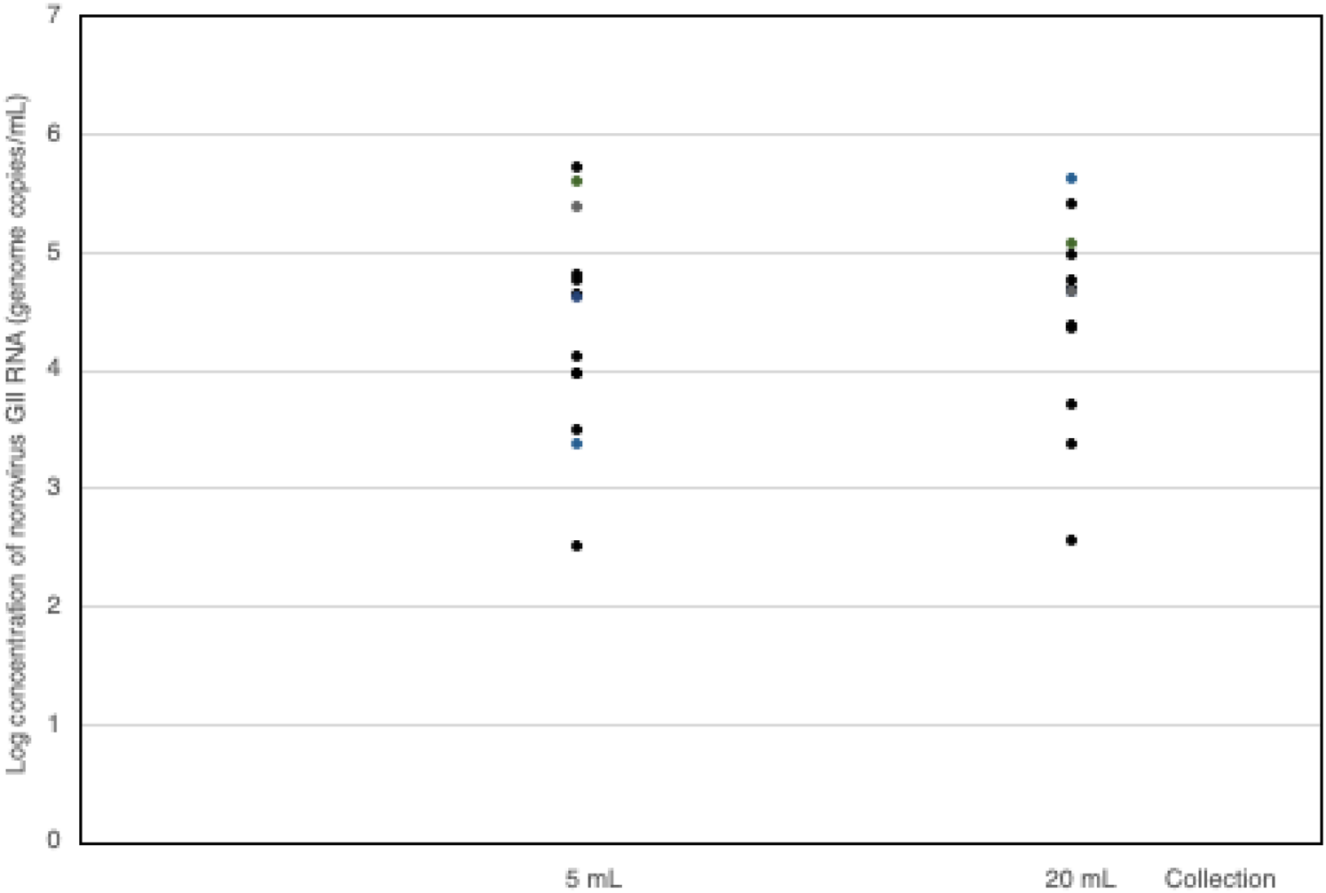
Quantification of norovirus GII RNA obtained in water concentrates from generated aerosols, collected by the SKC BioSampler in 5 and 20 mL water collection vessels using RT-qPCR

Trials were conducted to examine norovirus GII RNA concentrations in aerosols generated by the nebulizer, which contained norovirus GII at the concentrations of 10^3^ to 10^6^ genome copies/mL. The generated aerosols were collected using the SKC BioSampler and quantified for recovered norovirus GII. The aerosolized norovirus GII could be detected at concentrations in a range of 3.2 × 10^2^ − 5.2 × 10^5^ genome copies/mL for 5 mL and 3.7 × 10^2^ − 4.2 × 10^5^ genome copies/mL for 20 mL collection (Fig. 4). The lowest concentration of detected norovirus GII RNA was found to be 10^2^ genome copies/mL, whereas the maximum detectable concentration of norovirus GII RNA was 10^5^ genome copies/mL. The lowest concentrations of norovirus GII RNA for 5 mL and 20 mL collections were 3.2 × 10^2^ genome copies/mL or 8.6 × 10^2^ genome copies/m^3^ and 3.7 × 10^2^ genome copies/mL or 9.8 × 10^2^ genome copies/m^3^ of air volume, respectively. Norovirus GII RNA recoveries from the aerosolized samples were assessed in experiments of 5 mL and 20 mL collections. The average virus recovery of 5 mL collection was 25 ± 12% and that of 20 mL was 22 ± 19% in the established aerosol chamber. There was no significant difference in virus recovery of 5 and 20 mL collections (*p*-value = 0.310) (Table 1).

**Table 1:** Norovirus GII RNA recovery in the aerosol chamber using the nebulizer to produce aerosols and the SKC BioSampler to collect air sampled in water

Air sampling in water^a^	Norovirus GII RNA; Log genome copies^b^		Recovery (%)^b^	*P*-value
	Aerosolized by nebulizer	Obtained in SKC BioSampler		
5 mL collection	5.6 ± 1.1	5.0 ± 1.0	25 ± 12	0.310
20 mL collection	5.5 ± 0.7	4.7 ± 0.6	22 ± 19	

^a^at distance 36 cm and collected after aerosolization at a flow rate of 12.5 L/min, 15 min

^b^Mean ± Standard deviation

### RT-PCR Inhibition for Norovirus from Air Sampling

RT-PCR inhibitions for both noroviruses GI and GII were higher than the acceptable level (≤ 75%) with 5 and 20 mL aerosol control using undiluted RNA extract. The inhibition for norovirus GI was slightly higher than that for norovirus GII. However, after the RNA extract was diluted to 1:10 in nuclease-free water, the RT-PCR inhibitions were reduced to the level of ≤ 75%. There was significant difference in RT-PCR inhibition between the undiluted and 1:10 diluted RNA extract for norovirus GI (*p*-value =  < 0.001) and norovirus GII (*p*-value = 0.011) in both 5 mL and 20 mL collections (Table 2).

**Table 2:** RT-PCR inhibition of norovirus in aerosol control collection using the SKC BioSampler in a chamber

Air sampling in water	RT-PCR inhibition^a^					
	Norovirus GI		*P*-value	Norovirus GII		*P*-value
	Undiluted RNA	Diluted RNA 1:10		Undiluted RNA	Diluted RNA 1:10	
5 mL collection	90 ± 11	28 ± 9	< 0.001	80 ± 24	12 ± 9	0.011
20 mL collection	91 ± 15	28 ± 9		87 ± 12	24 ± 12	

^a^Mean ± Standard deviation; The acceptable level, ≤ 75%

## Discussion

Aerosolized droplets may play a role in the transmission of norovirus, causing acute gastroenteritis in various outbreaks (Alsved et al., 2020a; Bonifait et al., 2015; Zhang et al., 2017). We performed in vitro experiments with the generation of aerosols from a norovirus GII-positive stool sample using a high potency nebulizer under controlled laboratory conditions. The aerosols within an established chamber were collected in water using the SKC BioSampler and subsequently analyzed for norovirus GII RNA through RT-qPCR. Since this liquid impingement method collects the low density of virus, generally present in air samples, in a relatively large volume (20 mL) or less (5 mL) of liquid in the collection vessel, the dilution effect might impact norovirus detection. Thus, a concentrating process after aerosol sampling in water is needed to increase the possibility of virus detection. Various virus concentration methods, such as ultrafiltration (Uhrbrand et al., 2018), ultracentrifugation (Zheng et al., 2022), and conventional polyethylene glycol (PEG) precipitation (Lewis & Metcalf, 1988), have been used for environmental samples. While ultrafiltration and ultracentrifugation require sophisticated and expensive equipment, the virus recovery achieved by PEG precipitation in water samples is less than that with speedVac centrifugation, as used in our laboratory (Kittigul et al., 2001). SpeedVac centrifugation is effective in reducing sample volume, resulting in norovirus concentration from water samples (Kittigul et al., 2019). This concentrating method was employed in the present study to concentrate norovirus in aerosols collected in 5 and 20 mL samplings.

The distance between the nebulizer, used to generate aerosols, and the air sampler was found to impact virus recovery from bioaerosols (Brown et al., 2021). This study revealed that the recovery of viral RNA decreased as the SKC BioSampler was positioned farther from the nebulizer at distances of 4.5, 9.0, and 18 cm. Nevertheless, maintaining an appropriate distance, particularly at 36 cm from the nebulizer, demonstrated thorough dispersion of norovirus-containing aerosols throughout the chamber, resulting in a high recovery of norovirus GII RNA. Subsequently, the nebulizer placed at a 36 cm distance was used in further experiments since the dispersion of norovirus-containing aerosols at this distance might closely resemble the naturally occurring distribution of norovirus in aerosols.

Previous studies of murine norovirus as a surrogate for human norovirus addressed that norovirus might withstand aerosolization, suggesting a probable airborne transmission (Alsved et al., 2020b; Boles et al., 2020; Uhrbrand et al., 2018). Additionally, in norovirus outbreaks, the infected patients received norovirus via airborne transmission due to the possibility of norovirus’s ability to survive in air for a period of time (Marks et al., 2003; Xu et al., 2013). A strong association between norovirus-positive air samples and a 3-h time lapse since the last vomiting episode of the patients with acute gastroenteritis was demonstrated (Alsved et al., 2020a). It seems that norovirus can remain aerosolized in the air for at least 3 h. This small-scale study revealed that norovirus GII RNA either settled to the bottom of the chamber or was adsorbed to the interior wall within 120 min and 240 min post-nebulization for 5 and 20 mL collection, respectively. Different volumes of the collection vessels may influence the efficiency of virus collection in bioaerosols (Li et al., 2018).

A range of the viral load in aerosols that could be detected in water of 5 mL and 20 mL collections was consistent with a previous study that reported the concentration of murine norovirus (10^4^ genome copies/m^3^) detected in air sampling using the SKC BioSampler for 20 mL phosphate-buffered saline (Boles et al., 2020). After nebulization in the chamber, norovirus GII RNA as low as 10^2^ genome copies/m^3^ could be recovered by air sampling in water at 5 and 20 mL collections, exhibiting a highly sensitive procedure in the present study for norovirus detection in air samples. However, the results of RT-PCR inhibition suggest that detection of norovirus may underestimate the amount of virus in aerosols. PCR inhibitors, including polysaccharides, proteins, fats, RNases, metal ions, and others (Schrader et al., 2012) have been reported in air samples collected by passing large air volumes through filters (Luhung et al., 2018). The current study indicates the capability of RNA extract diluted at 1:10 in reducing PCR inhibitors to an acceptable level of RT-PCR inhibition (≤ 75%) and efficiently detecting norovirus RNA in aerosol sampling. Nevertheless, we found RT-PCR inhibition in both the nebulizer solution and the aerosols collected in water by the SKC BioSampler, exhibiting day-to-day variations which likely affected the reproducibility of aerosolized virus recovered in our experiments. The study’s limitations include the absence of measurements in the size range of aerosol particles and the quantification of norovirus GII RNA by RT-qPCR, representing both non-viable and viable virus. Viral culture method may be used to assess infectivity but is time-consuming and difficult to perform for human norovirus (Ettayebi et al., 2016). Further studies are needed to assess the infectivity of the virus and to reflect the typical conditions of naturally produced norovirus aerosols from infected patients with acute gastroenteritis. The present study suggests a use of SKC BioSampler for collection of aerosols in water, combined with speedVac centrifugation for virus concentration in the environmental monitoring of norovirus.

## Conclusion

This study presents the possibility of airborne transmission through norovirus GII-laden aerosols and provides quantitative data of the airborne dissemination in an aerosol chamber. The main advantage of using the protocol is the increased concentration of norovirus GII in water collected by the SKC BioSampler for air sampling. Norovirus GII is efficiently detected in the water concentrated by speedVac centrifugation from both 5 and 20 mL collections. RT-qPCR proves to be a sensitive molecular assay for detection and quantification of norovirus GII. This promising method can be used for surveillance of circulating noroviruses in air samples or other emerging respiratory viruses, contributing to prevention of health risks via aerosol transmission.

## Data Availability

All generated data during the current study are included in the article.
